# Interplay between Active Chromatin Marks and RNA-Directed DNA Methylation in *Arabidopsis thaliana*

**DOI:** 10.1371/journal.pgen.1003946

**Published:** 2013-11-07

**Authors:** Maxim V. C. Greenberg, Angelique Deleris, Christopher J. Hale, Ao Liu, Suhua Feng, Steven E. Jacobsen

**Affiliations:** 1 Department of Molecular, Cell and Developmental Biology, University of California Los Angeles, Los Angeles, California, United States of America; 2 Eli & Edythe Broad Center of Regenerative Medicine & Stem Cell Research, University of California Los Angeles, Los Angeles, California, United States of America; 3 Howard Hughes Medical Institute, University of California Los Angeles, Los Angeles, California, United States of America; University of California Riverside, United States of America

## Abstract

DNA methylation is an epigenetic mark that is associated with transcriptional repression of transposable elements and protein-coding genes. Conversely, transcriptionally active regulatory regions are strongly correlated with histone 3 lysine 4 di- and trimethylation (H3K4m2/m3). We previously showed that *Arabidopsis thaliana* plants with mutations in the H3K4m2/m3 demethylase *JUMONJI 14* (*JMJ14*) exhibit a mild reduction in RNA-directed DNA methylation (RdDM) that is associated with an increase in H3K4m2/m3 levels. To determine whether this incomplete RdDM reduction was the result of redundancy with other demethylases, we examined the genetic interaction of JMJ14 with another class of H3K4 demethylases: LYSINE-SPECIFIC DEMETHYLASE 1-LIKE 1 and LYSINE-SPECIFIC DEMETHYLASE 1-LIKE 2 (LDL1 and LDL2). Genome-wide DNA methylation analyses reveal that both families cooperate to maintain RdDM patterns. ChIP-seq experiments show that regions that exhibit an observable DNA methylation decrease are co-incidental with increases in H3K4m2/m3. Interestingly, the impact on DNA methylation was stronger at DNA-methylated regions adjacent to H3K4m2/m3-marked protein-coding genes, suggesting that the activity of H3K4 demethylases may be particularly crucial to prevent spreading of active epigenetic marks. Finally, RNA sequencing analyses indicate that at RdDM targets, the increase of H3K4m2/m3 is not generally associated with transcriptional de-repression. This suggests that the histone mark itself—not transcription—impacts the extent of RdDM.

## Author Summary

A number of factors contribute to the organization of eukaryotic genomes and the expression state of the underlying genes. For example, cytosine bases can be modified with the addition of a methyl-group. In the model plant *Arabidopsis thaliana*, methylated cytosines are typically associated with transcriptionally repressed regions—so called “heterochromatin.” Additionally, genomic DNA is wrapped around nucleosomes; each nucleosome consists of a complex of eight histone proteins. In turn, amino acid residues on histone proteins can be modified by a number of means, one of which is methylation. A methyl modification on lysine four of histone three (H3K4) is associated with transcriptional activation. Genome-wide studies in Arabidopsis have previously shown that DNA methylation and H3K4 methylation are highly anti-correlated. In this paper we examine a set of Arabidopsis mutants in which H3K4 methylation is abnormally high at a number of loci in the genome. At several of these loci, DNA methylation levels are decreased in the same mutants. These data suggest that H3K4 methylation antagonizes DNA methylation, which may contribute to mechanisms that distinguish active from silent regions of the genome.

## Introduction

Cytosine DNA methylation is an epigenetic mark that is conserved across all kingdoms of eukaryotes. Depending on its location in the genome, DNA methylation can be broadly classified as either genic or non-genic. Genic—or gene-body—methylation has been observed in several species of plants and animals, and generally correlates with transcriptionally active loci [1]–[3]. Conversely, non-genic methylation is associated with transcriptional repression at repetitive elements such as transposons [4]. Both plants and animals also have examples of non-genic DNA methylation repressing protein-coding gene transcription when the mark is present in the gene's regulatory regions [5], [6].

In the model plant *Arabidopsis thaliana*, gene-body methylation is exclusively found in the CG dinucleotide context and is maintained by METHYLTRANSFERASE 1 (MET1), the plant ortholog of mammalian DNA Methyltransferase 1 (DNMT1) [7]. In contrast, non-genic methylation is maintained by at least four methyltransferases: MET1, CHROMOMETHYLASE 2 (CMT2), CHROMOMETHYLASE 3 (CMT3), and DOMAINS REARRANGED METHYLTRANSFERASE 2 (DRM2). The four methyltransferases have distinct cytosine contexts that they preferentially act upon: CG (MET1), CHG (CMT3) and CHH (CMT2 and DRM2), where H is any base that is not a G [8], [9]. In addition to these well-characterized context preferences there is also a degree of redundancy for maintenance of non-CG methylation between CMT3 and DRM2 [8], [10], as well as CMT2 at some loci [9]. Furthermore, while all of the methyltransferases act in DNA methylation maintenance, only DRM2 is required for establishment of DNA methylation in all three sequence contexts [11].

In Arabidopsis, DNA methylation is correlated with specific histone marks that vary depending on the context and genomic location of the DNA methylation. Gene-body DNA methylation, for example, is largely co-incidental with histone 3 lysine 4 monomethylation (H3K4m1) [12]. Conversely, non-genic DNA methylation is strongly enriched in regions of histone 3 lysine 9 dimethylation (H3K9m2) [13]. Non-genic methylation is also inversely correlated with H3K4m2/m3—a mark that is associated with the 5′ ends of RNA Polymerase II (Pol II) genes [12], [14].

The link between H3K9m2 and CHG DNA methylation has been well established with regards to the CMT3 pathway: CMT3 binds to H3K9m2 through its eponymous chromodomain, as well as its bromo-adjacent homology (BAH) domain [15], [16]. Null mutant lines for the H3K9m2 histone methyltransferases recapitulate the *cmt3* DNA methylation phenotype, which illuminates the tight correlation between the two marks [7].

Links between histone modifications and the DRM2 pathway are also emerging. DRM2-dependent methylation depends on two plant specific RNA polymerases: RNA Polymerase IV and V (Pol IV and V). Pol IV generates a transcript that is processed into 24-nucleotide small interfering RNAs (siRNAs), and Pol V produces a transcript that serves as a scaffold for ARGONAUTE 4 (AGO4) loaded siRNAs that are generated by Pol IV [17], [18]. This dual-RNA polymerase system targets DRM2 to methylate DNA, although the specific mechanism for the targeting is not yet clear. Recent evidence suggests that Pol IV occupancy requires a factor, SAWADEE HOMEODOMAIN HOMOLOG 1 (SHH1), which is a dual histone modification sensor, preferentially binding to histones containing H3K9 methylation as well as lacking in H3K4 di- or trimethylation [19], [20].

We and others previously showed that mutation of the H3K4m2/m3 demethylase JMJ14 causes a partial reduction of DRM2-dependent RdDM, but does not affect the MET1 or CMT3 pathways [21], [22]. Since the observed decrease in DNA methylation correlated with a partial gain of H3K4 methylation, we concluded that H3K4m2/m3 might negatively impact RdDM. In this report, we tested whether the modest DNA methylation reduction phenotype of the *jmj14* mutant might be due to redundant activity of other histone demethylases. Arabidopsis contains a family of H3K4 demethylases distinct from JUMONJI proteins known as LYSINE-SPECIFIC DEMETHYLASE 1-LIKE (LDL). We show that mutation of two partially redundant members of the LDL family, *LDL1* and *LDL2*, causes a DNA methylation phenotype that is similar to *jmj14*, and that the *jmj14 ldl1 ldl2* triple mutant shows an enhanced methylation-loss phenotype. Interestingly, like the *jmj14* single mutant [21], the *jmj14 ldl1 ldl2* triple mutant reduced the maintenance of RdDM, but did not affect the establishment of DRM2-mediated methylation. Genomic analysis showed that the histone demethylase mutations only affect methylation at a subset of RdDM targets and that these targets are close to protein-coding genes. These results suggest that the JMJ14 and LDL histone demethylases reinforce RNA-directed DNA methylation near genes by counteracting nearby activating H3K4 epigenetic marks.

## Results

### *LDL1* and *LDL2* impact DRM2-mediated DNA methylation

We previously screened T-DNA insertional mutant lines in genes containing JmjC histone demethylase domains to determine whether perturbations in histone modifications might influence the establishment or maintenance of DNA methylation [21]. These results showed that mutation of the *JMJ14* gene reduced DRM2-mediated DNA methylation, but did not affect the MET1 or CMT3 pathways, and the effects were correlated with increased H3K4 di- and trimethylation. Interestingly, the DNA methylation reduction was not as strong as that observed in *drm2* mutants, suggesting the possibility that JMJ14 might be acting redundantly with other histone demethylases [21].

Lysine Specific Demethylase 1 (LSD1) is a well-characterized H3K4 demethylase in mammals [23], [24], and Arabidopsis contains four LSD1 homologs termed LDL1, LDL2, LDL3, and FLOWERING LOCUS D (FLD). Biochemical analysis suggests that LDL1 is exclusively an H3K4 demethylase with preference for mono- and dimethylation [25], and a previous report described LDL1 and LDL2 as partially redundant H3K4 demethylases that reduced DNA methylation at the *FLOWERING WAGENINGEN* (*FWA*) gene [26]. It should be noted however that, even though mammalian LSD1 only demethylates H3K4 *in vitro*, it has both H3K4 and H3K9 demethylase activity *in vivo* [23], [27]. Thus we cannot rule out the possibility that LDL1/LDL2 may have more diverse biological functions *in planta*. We observed that the *jmj14-1* mutant shows reduced DNA methylation and increased H3K4 methylation at *FWA* to about the same degree as that reported for *ldl1-2 ldl2* double mutants [21], [26]. To study possible genetic interactions between the two families of demethylases, we generated a *jmj14-1 ldl1-2 ldl2* triple mutant line.

**Figure 1:**
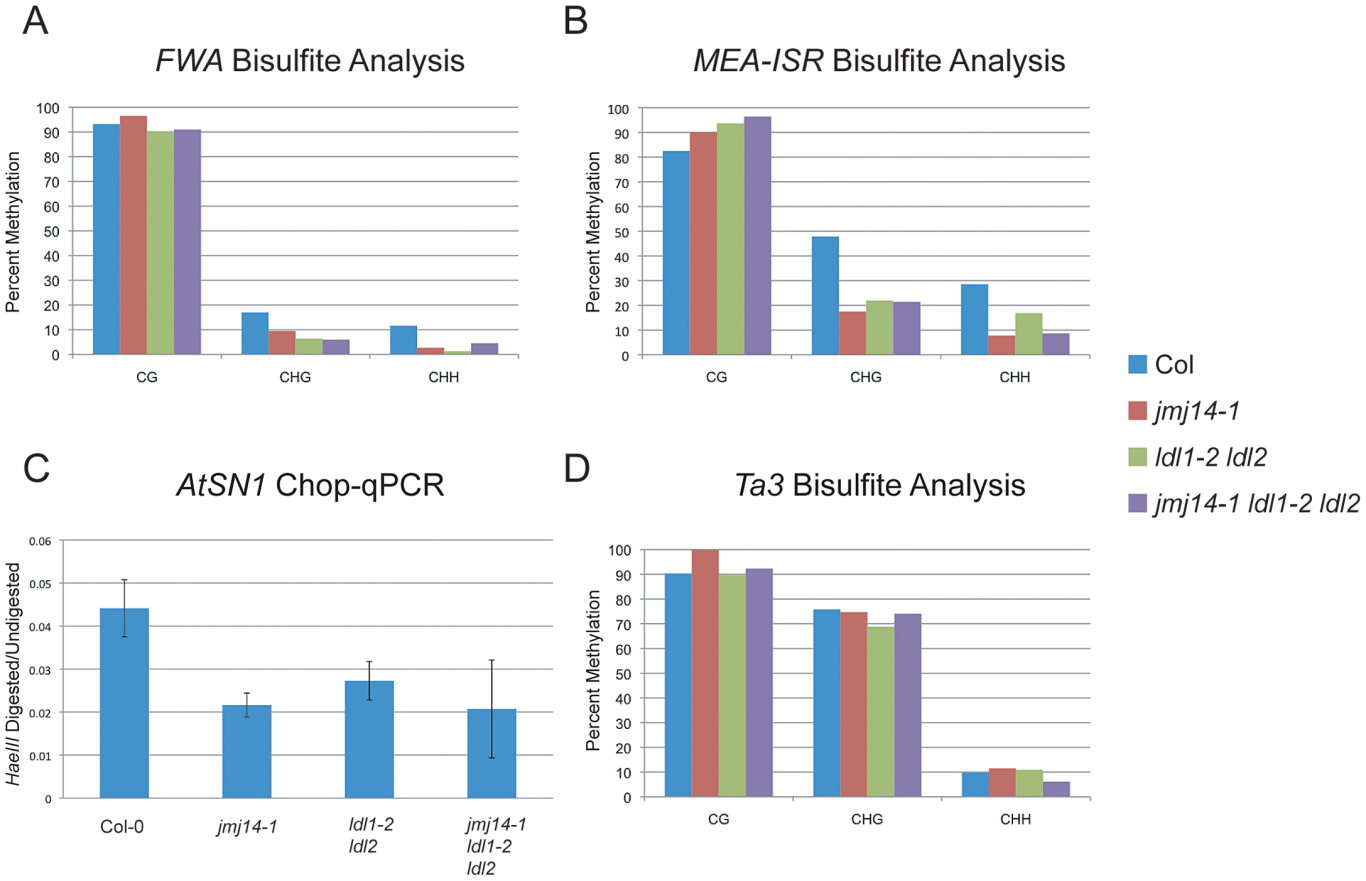
DNA methylation of RdDM targets in histone demethylase mutants. (A) Bisulfite analysis of the *FWA* endogene. (B) Bisulfite analysis of the *MEA-ISR* tandem repeats. (C) *AtSN1* Chop-qPCR. Genomic DNA was digested with *HaeIII*, which recognizes GGCC sites, but is sensitive to cytosine that is methylated. In the region amplified, there are three *HaeIII* sites, all corresponding to asymmetric cytosine contexts. The signal is relative to undigested DNA for each genotype. (D) Bisulfite analysis of the *Ta3* transposon.

Consistent with the data reported by Jiang et al., we did observe an increase in H3K4 dimethylation and trimethylation (m2/m3) in the *ldl1-2 ldl2* double mutant (Figure S1A) [26]. However, while Jiang et al. reported a CG methylation defect at the *FWA* repeats, we did not observe any such effect by bisulfite sequencing analysis (Figure 1A). Rather, we only observed a reduction in non-CG methylation that is much more similar to that observed in *jmj14-1* (Figure 1A). Consistent with a defect in RdDM, this same study did show a reduction in non-CG methylation at the *FWA* transgene in *ldl1 ldl2* double mutants [26]. In analysis of other RdDM targets, we observed a similar phenomenon. At the *MEDEA-INTERGENIC SUBTELOMERIC REPEATS* (*MEA-ISR*), there was no reduction in MET1-dependent CG methylation, and a decrease in non-CG methylation, once again, similar to that observed in *jmj14-1* (Figure 1B). We also analyzed the *AtSN1* transposon using a quantitative PCR (qPCR) based assay in which we digested genomic DNA with the *HaeIII* endonuclease that is sensitive to CHH methylation at three restriction sites within the amplified region (Figure 1C). We observed increased digestion in the *ldl1-2 ldl2* double mutant, the *jmj14-1* single mutant, and the *jmj14-1 ldl1-2 ldl2* triple mutant. We also analyzed the *Ta3* locus by bisulfite sequencing (Figure 1D). *Ta3* is methylated by MET1 and CMT3, but not DRM2 [8]. Similar to *jmj14-1*, the *ldl1-2 ldl2* double mutant showed no impact on *Ta3* methylation.

### Histone demethylases do not impact DNA methylation establishment

Prior to our initial study describing JMJ14, all mutations that caused a reduction in the maintenance of RNA-directed DNA methylation were also found to be required for the establishment of DNA methylation [28]. In order to examine the requirements of DNA methylation establishment, we take advantage of a transgenic version of the *FWA* gene. *FWA* is a homeodomain transcription factor with tandem repeats in its 5′ UTR. In unmethylated epialleles, the *FWA* gene is ectopically expressed, causing a delay in flowering time [29]. Unmethylated transgenes introduced into wild-type plants are recognized by the RdDM machinery, and methylated and silenced [11], [30]. However, in mutants such as *drm2* that are unable to establish DNA methylation, transgenic *FWA* expression leads to late flowering.

Previous *FWA* transformation assays on the *jmj14-1* mutant showed the surprising result that flowering time and DNA methylation establishment were not affected [21], even though *jmj14-1* reduces the maintenance of RdDM at the *FWA* locus. To test whether the other histone demethylase gene mutations might affect *de novo* methylation of *FWA*, we transformed *ldl1-2 ldl2* and *jmj14-1 ldl1-2 ldl2* with *FWA* and scored for flowering time (Figure S2). Despite previously published evidence that LDL1 and LDL2 were required for DNA methylation establishment, we observed that each untransformed mutant line exhibited a flowering-time phenotype that deviated only slightly from wild type [26], [31]–[33]. More importantly, the flowering time after *FWA* transformation was comparable to the slight delay also observed in wild-type Col-0 plants, showing that none of the mutations caused a block in *de novo* silencing of *FWA*. However, one cannot completely exclude the possibility that de-repression of *FLOWERING LOCUS T* (*FT*) in the *jmj14* mutant background [31]–[33] may partially offset ectopic *FWA* expression. While it is not clear why the histone demethylase mutations cause decreases in the maintenance of RdDM but do not affect methylation establishment, it is possible that the nature of chromatin at the time of DNA methylation establishment (early zygotic development [34]) is such that histone demethylases are not required at this particular stage.

### Histone demethylase mutants show losses of RNA-directed DNA methylation at only a subset of loci

**Figure 2:**
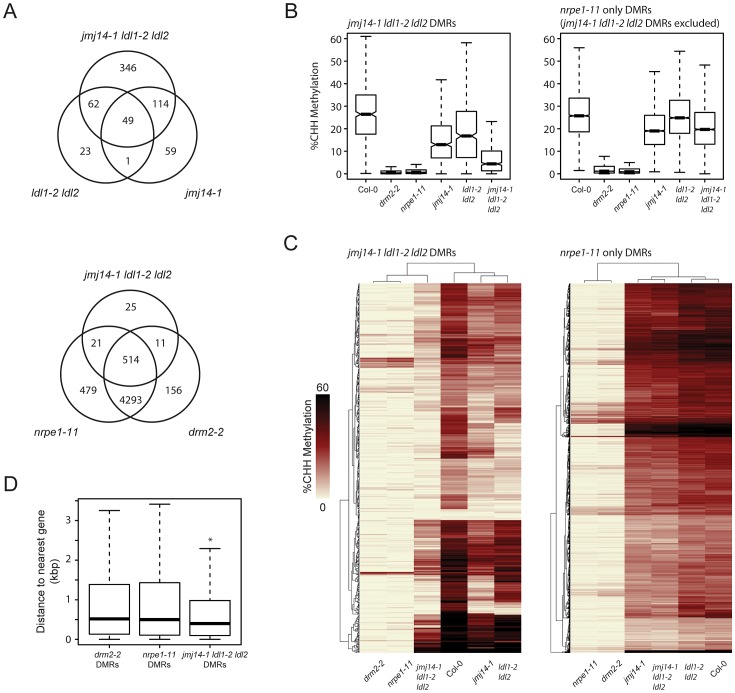
De novo identification of DMRs in *jmj14-1 ldl1-2 ldl2* and *nrpe1-11* mutant backgrounds. (A) Venn diagrams representing the genomic overlap of DMRs identified in each mutant background. (B) Boxplot of CHH methylation levels at DMRs of given groups. (C) Distance clustered heat map of CHH methylation levels at DMRs of listed groups. (D) Boxplot of distances between DMRs of listed mutant backgrounds and the nearest protein-coding gene. * indicates a significant difference from the *drm2* and *nrpe1* DMRs (P<2.2e-14, Welch Two Sample t-test).

To ascertain the global impact on DNA methylation of the various histone demethylase mutants, we performed whole genome shotgun bisulfite sequencing (BS-Seq). We analyzed the data in parallel with those generated from *drm2-2* mutants and *nrpe1-11* mutants (the largest subunit of Pol V) in order to draw a direct comparison with canonical RdDM factors [10]. Since the RdDM pathway primarily impacts CHH methylation, we defined differentially methylated regions (DMRs) for CHH context methylation in each mutant (Table S1; Figure 2). Although JMJ14 and LDL1/LDL2 appear to have some preferential targets, there was a large degree of overlap observed by comparing their respective DMRs. Moreover, the greatest number of DMRs appear in the triple mutant, suggesting that JMJ14 and LDL1/LDL2 act in a mostly redundant fashion to maintain DNA methylation (Figure 2A).

To determine whether the histone demethylase mutants have a generally weak DNA methylation defect at all RdDM sites or if they might be acting more strongly at only a subset of RdDM targets, we assayed CHH methylation levels at DMRs defined in the demethylase mutants and compared these with DMRs in a strong RdDM mutant, *nrpe1-11* (Figure 2A and B). We observed a strong loss of CHH methylation in the demethylase mutants which was enhanced in the triple mutant. However, we observed only a slight loss of CHH methylation in the demethylase mutants at the bulk of canonical RdDM sites (Figure 2A and B), suggesting that demethylases do not serve as general effectors of RdDM, but act in a locus specific manner. Furthermore, we found that the DMRs in the *jmj14-1 ldl1-2 ldl2* triple mutant overwhelmingly overlapped with the *nrpe1-11* DMRs (Figure 2A), strongly suggesting that DNA methylation defects in the histone demethylase mutants are mainly limited to RdDM targets.

We further analyzed the CHH methylation defects in the histone demethylase mutants by heat-map analysis of the DMRs. As expected, *drm2-2* mutants exhibited virtually a complete loss of CHH methylation at all NRPE1 sites (Figure 2C). Consistent with our other analyses, the histone demethylase mutants uniformly displayed a much more minor methylation loss at most sites. Conversely, at DMRs defined in *jmj14-1 ldl1-2 ldl2*, there was a dramatic loss in CHH methylation in *drm2-2* and *nrpe1-11* (Figure 2C). In addition, at the histone demethylase DMRs, there were many examples of synergistic effects between the *jmj14-1* single mutant and the *ldl1-2 ldl2* double mutant, with a loss of CHH methylation only apparent in the *jmj14-1 ldl1-2 ldl2* triple mutant (Figure 2C). Taken together, these data strongly suggest that histone demethylases act in a partially redundant fashion to control RNA-directed DNA methylation at a subset of loci.

To determine the nature of the histone demethylase mutant DMRs and to see if they had unique characteristics compared to DMRs in canonical RdDM mutants, we further analyzed them relative to annotated genes, based on our initial observations at individual loci that suggested that they tended to be closer to protein-coding genes. When we calculated the distance of the DMRs to protein-coding genes, we found that the average distance is significantly smaller for *jmj14-1 ldl1-2 ldl2* DMRs than for *drm2* or *nrpe1* DMRs (Figure 2D, Figure S3). It is known that JMJ14 and LDL1/LDL2 regulate non-DNA methylated protein-coding genes through their H3K4 demethylase activity [21], [26], [31]–[33]. Thus a likely explanation for these results is that transposons or other silent elements in proximity to actively H3K4-demethylated genes might be more sensitive to mutations in H3K4 demethylases than other sites.

### JMJ14 and LDL1/LDL2 act largely additively at RdDM sites

Given that two families of histone demethylases appear to functionally overlap at a large number of RdDM targets, we sought to use our sets of genomic data to better understand their genetic interaction. Our DMR identification analysis suggests that JMJ14 and LDL1/LDL2 have some sites of preferential action (Figure 2A). However, further analyses of these data indicate that at a majority of identified demethylase DMRs, both the *jmj14-1* single mutant and the *ldl1-2 ldl2* double mutants reduce CHH methylation to varying degrees that are enhanced in the *jmj14 ldl1-2 ldl2* triple mutant (Figure S4). Thus the number of overlapping regions of DNA hypomethylation for the histone demethylases reported in Figure 2A is likely an underestimate, as some regions were omitted for not meeting our thresholds for calling DMRs in a given genotype, yet still show a subtle methylation effect in that genotype. In addition, these data indicate a high degree of overlap in the sites affected in *jmj14* and *ldl* mutants and, given the general enhancement seen in the triple mutant, suggest that these demethylases act in a largely additive fashion with regards to RdDM sites in the genome. The exceptions to this interpretation are those small number of DMRs unique to either the *jmj14-1* or *ldl1-2 ldl2* genotypes which show CHH methylation defects that are not strongly enhanced in the demethylase triple mutant (Figure S4). This suggests a low level of locus-specific preference for either class of demethylase that is as yet not understood. In mammals, it is known that LSD1 can exist in both the CoREST and NuRD repressive complexes in which they perform different activities [35], [36]. Similarly, LDL1/LDL2 and JMJ14 could exist in different complexes depending on the locus of action, and thus have differential effects on DNA methylation in a locus-specific manner. This possibility will deserve further investigation in future studies.

### Decreases in DNA methylation in *jmj14-1 ldl1-2 ldl2* are accompanied by an increase in H3K4 methylation

In order to further understand the relationship between H3K4 methylation and DNA methylation at RdDM targets, we performed chromatin immunoprecipitation (ChIP) against H3K4 methylation marks in the suite of histone methylation mutants. At three individual loci analyzed, *FWA*, *MEA-ISR*, and *AtSN1*, we observed only moderate gains in H3K4m2/m3 in the demethylase mutants (Figure S1). A comparison of these data with the DNA methylation data at these three loci (Figure 1) suggests that even slight increases in H3K4m2 or H3K4m3 are associated with reduced RNA-directed DNA methylation at these sites.

**Figure 3:**
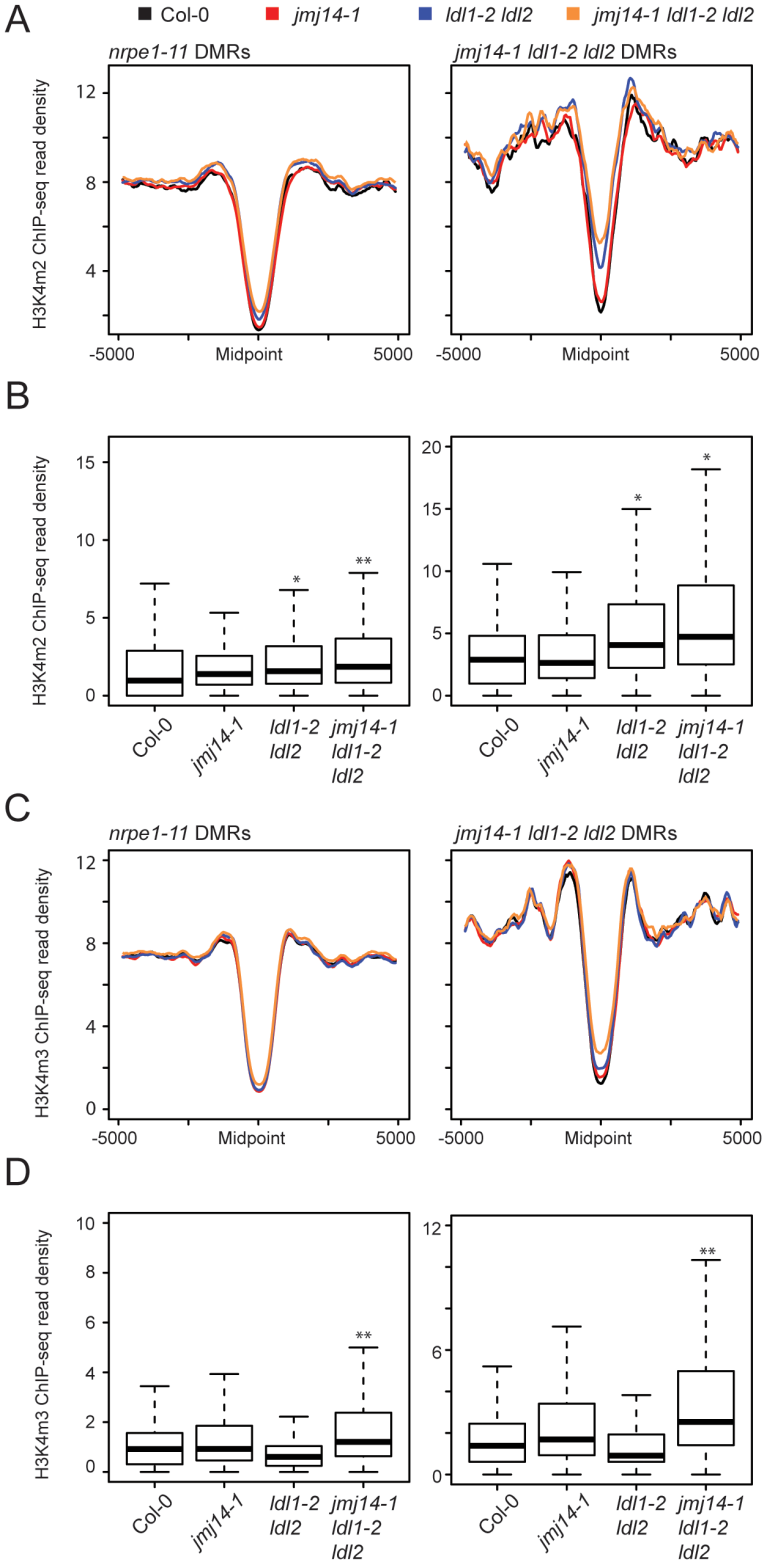
Global H3K4m2/m3 ChIP analysis. Metaplots (A) and boxplots (B) of H3Km2 ChIP-seq read density (RPKM) over DMR groups in demethylase mutant genotypes. For boxplots, DMRs were considered as the 1000 bp region extending +/−500 bp from the DMR midpoint. * indicates a significant gain in read density for a given library relative to wild type (P<1e-15, Mann-Whitney U Test) and ** indicates a gain in read density relative to all other libraries including wild type (P<1e-15, Mann-Whitney U Test). (C) and (D) present similar analyses for H3K4m3 ChIP-seq libraries.

To gain a more global perspective on the relationship between DNA methylation and H3K4 methylation, we performed ChIP against H3K4m2 and H3K4m3 marks coupled with high-throughput sequencing (ChIP-seq). Across DMRs defined in *nrpe1-11* and *jmj14-1 ldl1-2 ldl2*, H3K4 di- and trimethylation were depleted (Figures 3A). This result was expected given a previous study demonstrating that H3K4 di- and trimethylation and DNA methylation are anti-correlated [12]. In addition, in the *jmj14-1 ldl1-2 ldl2* triple mutant we observed an increase of H3K4 di- and trimethylation specifically at DMRs defined from the *jmj14-1 ldl1-2 ldl2* triple mutant BS-seq data (Figures 3A and C, Figure S5). These results show that genomic regions experiencing the largest alterations of DNA methylation levels in the histone demethylase triple mutant mutants generally showed the largest increase in H3K4m2/m3. Although we did see some increases in H3K4m2 and H3K4m3 in the *jmj14-1* and/or in the *ldl1-2 ldl2* mutants, the affects were more variable and were not as strong as those seen in the *jmj14-1 ldl1-2 ldl2* triple mutant (Figure 3B and D).

We also wanted to rule out the possibility that the increase in H3K4 methylation might be an indirect result of DNA hypomethylation. Therefore we also performed H3K4m2 and H3K4m3 ChIP-seq in *drm2-2, nrpe1-11*, as well as an upstream RdDM mutant *nrpd1-4* (the largest subunit of Pol IV), and analyzed the H3K4 methylation profile at both *nrpe1-11* and *jmj14-1 ldl1-2 ldl2* DMRs (Figure S6). While the loss of CHH methylation at these DMRs is dramatic in *nrpe1-11* and in *drm2-2* mutants (Figure 2B), there was little increase of H3K4 methylation in these mutants, but significant gain of methylation in the triple histone demethylase mutant (Figure S6). Therefore, we conclude that the increase in H3K4 methylation antagonizes RdDM, and not vice versa.

Finally, these ChIP data provide further insight into the nature of the small number of specific DMRs found in the histone demethylase mutants compared to canonical RdDM factors (Figure 2A). As we showed above, the DMRs in the triple mutant are on average closer to genes (Figure 2D). Consistent with this, we observed a higher level of H3K4 methylation flanking the midpoint of DMRs defined in *jmj14-1 ldl1-2 ldl2* mutants than in those defined in *nrpe1-11* (Figure 3A and C). This was true in wild-type plants and demethylase mutants, showing that the *jmj14-1 ldl1-2 ldl2* DMRs are in regions that are closer to highly H3K4 methylated areas, which are primarily represented by protein-coding genes. These data suggest that JMJ14 and LDL1/LDL2 act at a specific subset of RdDM targets to prevent accumulation of H3K4 methylation in silent regions, which would otherwise antagonize the DNA methylation machinery. In more gene-poor regions, histone demethylases would not be required, thus the mutants do not display a DNA methylation phenotype.

### Histone demethylase mutants do not cause transcriptional de-repression at DMRs

**Figure 4:**
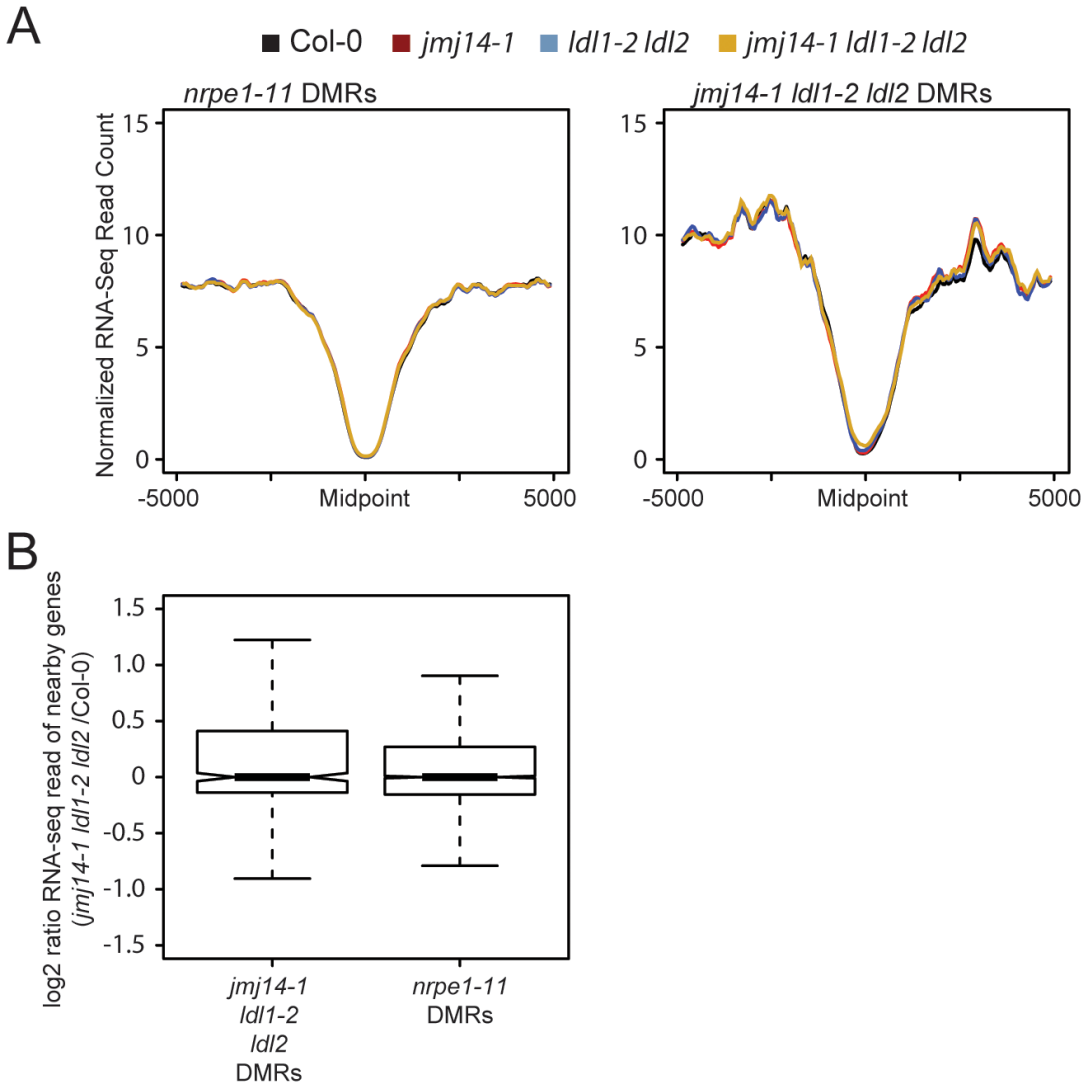
Changes in histone methylation at DMRs do not correlate with alterations in mRNA expression. (A) Metaplot of normalized RNA-seq read counts over *nrpe1-11* and *jmj14-1 ldl1-2 ldl2* DMRs. (B) log2 ratio of gene expression for genes with promoters overlapping with different DMRs.

Even though a majority of RdDM targets are not over genic regions but are instead upstream of genes in promoter regions, we reasoned that one possible explanation for the gain of H3K4 at demethylase DMRs would be aberrant transcription at these sites in the demethylase mutants. Indeed, many RdDM targets are transposable elements that may be competent for transcription in a demethylase mutant background. Such aberrant transcription by Pol II could potentially displace Pol IV or Pol V activity, resulting in reduced DNA methylation targeting. To test the hypothesis that demethylase mutant effects on RdDM were caused indirectly by a transcription-based mechanism, we performed a comparative transcriptome analysis by RNA sequencing (RNA-seq) between wild type and mutants (Figure 4). Strikingly, we observed no increase in transcription at DMRs defined in either *nrpe1-11* or in *jmj14-1 ldl1-2 ldl2* (Figure 4A). In addition, we did not observe increased transcription of protein-coding genes nearest the defined DMRs (Figure 4B). Thus the increase of H3K4 methylation in the proximity of DNA methylated regions does not affect transcriptional activity of these regions. Together, these data indicate that it is not transcription *per se* that affects RdDM, but more likely the nature of the chromatin itself.

### sRNA accumulation is decreased at histone demethylase mutant DMRs

**Figure 5:**
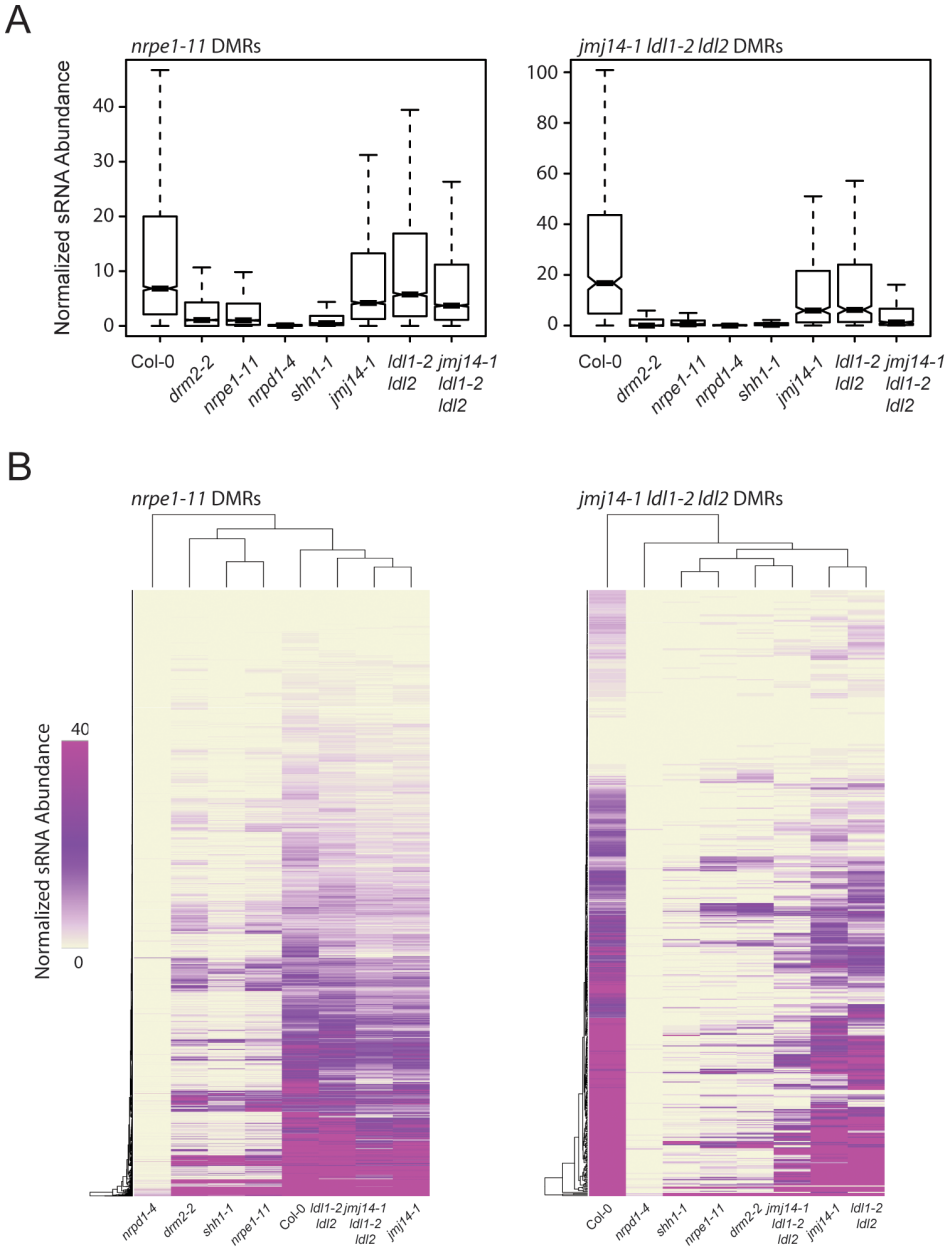
*jmj14-1 ldl1-2 ldl2* triple mutants are deficient for sRNA production at affected DMRs. (A) Boxplots of 24-nt siRNA abundance (RPKM) at *nrpe1-11* and *jmj14-1 ldl1-2 ldl2* DMRs for various mutants. (B) Distance clustered heat map of 24-nt siRNA abundance at DMRs for listed genotypes.

RdDM is an siRNA-driven process and previous studies have shown that siRNA profiles in different DNA methylation mutants can help position these mutations in the RdDM pathway. We tested whether mutations in H3K4 demethylases impacted siRNA accumulation by generating small RNA (sRNA) libraries and performing high-throughput sequencing (sRNA-seq). At DMRs defined in *nrpe1-11*, there was only a very small decrease in sRNA levels in the three histone demethylase mutant lines analyzed (Figure 5A). However, there was a marked reduction of sRNA levels at *jmj14-1 ldl1-2 ldl2* DMRs (Figure 5A). Heat map analysis similarly showed that at the great majority of DMRs from the *jmj14-1 ldl1-2 ldl2* triple mutant, there was depletion of sRNA reads, and that at many DMRs, there was nearly a complete loss of sRNAs (Figure 5B).

Mutations in certain RdDM components have been shown to exhibit locus specific reductions of siRNAs, including mutation in *SHH1*, as well as mutation of downstream RdDM factors such as *DRM2* and *NRPE1* [20]. *SHH1* encodes a factor that facilitates recruitment of Pol IV to a subset of loci, and *shh1* mutants show losses of siRNAs at largely the same subset of loci as do *drm2* and *nrpe1* mutants [20]. To analyze whether there is an overlap between the loci affected in these other RdDM components and the histone demethylase mutations, we included in our analysis sRNA-seq data from *shh1-1, drm2-2*, and *nrpe1-11*, as well as *nrpd1-4*, which is a mutation in the largest subunit of Pol IV (Figure 5A). We observed a nearly total loss of siRNAs from the *jmj14-1 ldl1-2 ldl2* triple mutant DMRs in all the RdDM mutants tested (Figure 5A), indicating these sites require SHH1 and downstream RdDM factors for normal RdDM pathway function and suggesting that a common mechanism may regulate siRNA levels at these sites.

## Discussion

In this study we have described the relationship between H3K4 methylation and DRM2-mediated DNA methylation through the study of histone demethylase mutants. Genome-wide ChIP and bisulfite sequencing analyses show that H3K4m2 and H3K4m3 marks antagonize the RdDM pathway at a large number of sites in the genome, and these sites are enriched near the 5′ end of K4-methylated protein-coding genes, which suggests that the activity of histone demethylases is more crucial for DNA methylation integrity nearby H3K4m2/m3 rich promoters. Our transcriptome data also indicate that H3K4m2 and H3K4m3 marks *per se*, and not associated transcriptional changes, act to prevent complete RNA-directed DNA methylation.

Together, these data raise interesting questions about the mechanisms by which active chromatin marks like H3K4 methylation may affect the stable maintenance of repressive DNA methylation. In mammals, Dnmt3L—the binding partner of the *de novo* methyltransferase Dnmt3A—specifically binds to unmodified H3K4 (H3K4m0) [37], [38]. Although it cannot be ruled out that a convergent mechanism evolved in plants, there is no evidence for such a relationship between H3K4m0 and DRM2, and DRM2 lacks the Plant Homeodomain (PHD) found in Dnmt3L that recognizes H3K4m0. Because regions that lose methylation in *jmj14 ldl1 ldl2* triple mutants also showed a reduction in siRNA levels, we propose that SHH1 recruitment of Pol IV may be a link that can explain the effect of histone demethylases on RNA-directed DNA methylation. SHH1 interacts with Pol IV, and is required for 24-nt siRNA biogenesis at a large subset of RdDM targets [19], [20], including at those sites that lose methylation in *jmj14-1 ldl1-2 ldl2* triple mutants (Figure 5A). Structural and biochemical data indicate that SHH1 binds to H3K9m2 through its tandem-tudor-like SAWADEE domain. Interestingly, SHH1 is also inhibited from binding to histone tails *in vitro* when H3K4m2/m3 is also present. Therefore, we propose that the DNA methylation defect observed in H3K4 demethylase mutants may be due to impaired SHH1 binding, reduced Pol IV recruitment, and reduced siRNA biogenesis. In this way, JMJ14 and LDL1/2 can serve to reinforce methylation of silent transposons and other repeated sequences that are nearest to protein-coding genes.

## Materials and Methods

### Plant material

All plants utilized in this study are in the Col-0 ecotype, and grown under long day conditions. The following mutant lines were used: *jmj14-1* (SALK_135712), *ldl1-2* (SALK_034869), *ldl2* (SALK_135831), *drm2-2* (SALK_150863), *nrpd1-4* (SALK_08305), *nrpe1-11* (SALK_02991), and *shh1-1* (SALK_074540C).

### Data submission

All whole-genome sequencing datasets were submitted to the Gene Expression Omnibus (GEO) database and are accessible as part of the GSE49090 accession.

### Bisulfite sequencing and analysis

For sodium bisulfite sequencing, DNA was treated using the EZ DNA Methylation Gold kit (Zymo Research) by following the manufacturer's instructions. Amplified PCR fragments from each analyzed locus were cloned into pCR2.1-TOPO (Invitrogen) and sequenced. We analyzed 15 to 22 clone sequences per sample using Lasergene SeqMan software. In order to distinguish the *FWA* transgene from the endogene, we destroyed a *BglII* restriction site in the transgenic copy in the region of PCR amplification. We then bisulfite treated genomic DNA of transgenic plants following a *BglII* digestion (37°C, overnight), which prevented amplification of the endogenous gene. Additionally, the transgenic copy of *FWA* was derived from the Landsberg ecotype, thus we could distinguish between the transgene and endogene based on the existence of three single nucleotide polymorphisms within the amplicon in case *BglII* digestion was not complete. Primers used for amplification are listed in Table S2.

### qPCR-Chop assay

Analysis of asymmetric methylation at the *AtSN1* locus was performed exactly as described in [21]. Primers used for amplification are listed in Table S2. Analysis of non-CG methylation at *AT5G35935* was performed by extracting DNA from young flowers using a standard Cetyl trimethyl ammonium bromide protocol. A total of 200 ng of genomic DNA was digested overnight at 37°C with *MspI* side-by-side with samples containing buffer and no enzyme (undigested). Quantitative real-time PCR validation of uncut DNA after *MspI* digestion was performed using the Bio-Rad Synergy Brands Green SuperMix on an MX3000 Stratagene cycler. The PCR parameters are as follows: one cycle of 10 min at 95°C, 40 cycles of 30 s at 95°C, 1 min at 55°C, and 1 min at 72°C. PCR primers sequences are listed in Table S2.

### Generation of transgenic plants

Transgenic plants were generated as described in [39].

### Flowering time

We measured flowering time of plants as the total number of leaves (rosette and cauline leaves) developed by a plant at the time of flowering. Plants transformed with the *FWA* transgene were selected for by spraying with a 1∶1000 dilution of Basta soon after germination.

### Chromatin immunoprecipitation

ChIP assays were performed as described in [40] with modifications. For immunoprecipitation, the following antibodies were used: H3K4m2, Abcam AB32356; H3K4m3, Diagenode pAb-003-050. Primers used for amplification of ChIP targets are listed in Table S2.

### Genome-wide mRNA sequencing

Total RNA was prepared using a standard Trizol extraction from 0.5 grams of 3-week-old plant aerial tissue. 4 µg of total RNA was then used to prepare libraries for Illumina sequencing, following the Illumina TruSeq RNA Sample Prep guidelines. Multiplexed samples were sequenced at 50-nt length on an Illumina HiSeq 2000 instrument.

### Genome-wide ChIP and library generation

ChIP of the Col, *jmj14*, *ldl1 ldl2*, and triple mutants shown in Figure 3 was performed as described above. Libraries were generated as described in [41]. For the ChIP-seq analysis of RdDM mutants done in parallel with Col and the demethylase triple mutant (Figure S6), ChIP was carried out as described in [20] using 10-day-old seedlings and the following antibodies: H3K4m2, Millipore 07-030; H3K4m3, Millipore 04-745. Subsequent ChIP-seq libraries were generated as described in [20].

### Shotgun bisulfite sequencing

Genomic DNA was extracted from one gram of 3-week-old plant aerial tissue using a DNeasy Plant Maxi Kit (Qiagen). Libraries for bisulfite sequencing were generated and sequenced as described in [42], with the change that sequencing was carried out on an Illumina HiSeq 2000 instrument.

### sRNA purification and library generation

Small RNAs were purified from Trizol-purified total RNA by fractionation with one volume of 25% PEG 8000, followed by gel purification of ∼15–30-nt RNA species from a 20% polyacrylamide gel (7M urea). The initial total RNA was isolated from 100 mg of immature floral buds, and the resulting siRNA fraction was resuspended in 10 uL TE buffer, all of which was used for library generation. sRNA-seq libraries were generated using the small RNA TruSeq kit (Illumina) following the manufacturer instructions with the exception that 15 cycles were used during the amplification step.

### Data analyses

Sequenced reads were base-called using the standard Illumina pipeline. For ChIP-seq, mRNA-seq and BS-seq libraries, only full 50-nt reads were retained. For sRNA-seq, reads had adapter sequence removed and were retained if between 18 and 28-nt in length. For ChIP-seq and mRNA-seq, only uniquely mapping reads, allowing for 1 mismatch, were mapped to the Arabidopsis genome (TAIR10 – www.arabidopsis.org) with Bowtie [43] and retained for further analysis. For sRNA-seq, both unique and non-unique reads were mapped to the genome, allowing for one mismatch, and for downstream read density calculations, only the unique reads were considered with the total reads (unique+non-unique) being used for normalization purposes between libraries. For BS-seq libraries, reads were mapped using the BSseeker wrapper for Bowtie [44]. For ChIP-seq, mRNA-seq, and BS-seq, identical reads were collapsed into one read to avoid optical or PCR based duplicates. For the calculation of read density in sRNA-seq libraries where duplicate reads may be of biological significance, up to 100 identical reads were retained as distinct reads in any given region, with any reads exceeding that flattened to the 100 read cap.

For methylation analysis, percent methylation was calculated as described [10]. DMRs were defined using the bsseq package of the R-based BSmooth pipeline [45]. For the purposes of defining CHH hypomethylated DMRs, only cytosines with 2× coverage in a majority of the wild-type libraries as well as the mutant library in question were utilized after data smoothing. Variances were estimated from the wild-type library group, and initial identified DMRs were filtered for t-statistics <−2 or >2. These filtered DMRs were then further filtered by keeping only those covering > = 20 assayed CHH context cytosines and a mean difference > = 0.1. Finally, the DMRs were filtered once more and only those with an area statistic  = <−100 or > = 100 were retained for the final set of DMRs. For each mutant genotype the corresponding BS-seq library was compared to four wild-type (ecotype Col-0) libraries. One of these wild-type libraries is submitted as part of the current study GEO record (GSE49090), while the other 3 wild-type replicates were previously published and are thus part of other GEO records (GSE39901 – listed as “WT replicate 2”; GSE38286 ; GSE36129). For Figure 2, the wild type represented is the Col-0 replicate from the GSE36129 study as these plants were grown side-by-side with the demethylase mutants.

For all libraries and analyses, the list of mRNA used, along with genomic coordinates, were obtained from TAIR (TAIR10). For all analyses, overlap was considered to > = 1 bp overlap of defined regions. All statistical analysis was conducted within the R environment.

## Supporting Information

Figure S1 H3K4m2 and H3K4m3 ChIP-qPCR analysis of RdDM targets in histone demethylase mutants. The analysis was performed at *FWA* (A), *MEA-ISR* (B), and *AtSN1* (C). Data were normalized to input DNA and to an internal control (*ACTIN*). The average of three independent ChIP-experiments is shown (for each experiment, qPCRs were performed in duplicate).
(TIF)

Figure S2 *FWA* methylation establishment assay and flowering-time analysis in chromatin effector mutants. Flowering-time is determined by the total number of rosette and cauline leaves when the first inflorescence appears. *FWA* transformed lines are compared to untransformed lines of the same genotype. The graph depicts averages from populations of >20 individual plants.
(TIF)

Figure S3 Relative abundance of mutant CHH DMRs at and around the transcription start site (TSS) of protein-coding genes. Relative abundance is calculated as ((average coverage of DMRs over gene region)/total number of thousands of DMRs).
(TIF)

Figure S4 Analysis of subsets of DMRs defined in the demethylase mutants. Subsetting the demethylase DMRs identified in Figure 2A reveals some sites of preferential activity by either class of demethylase in regulating CHH methylation levels with a general trend of an enhanced CHH defect in the *jmj14-1 ldl1-2 ldl2* triple mutant. * The single DMR represented as the union of *jmj14-1* and *ldl1-2 ldl2* DMRs to the exclusion of *jmj14-1 ldl1-2 ldl2* DMRs was not plotted given the limited data in a single data point.
(TIF)

Figure S5 Correlation of H3K4 methylation changes and CHH context DNA methylation at *jmj14-1 ldl1-2 ldl2* triple mutant CHH DMRs. Correlation of weighted change in H3K4m2/H3K4m3 and CHH methylation in *jmj14-1 ldl1-2 ldl2* at *jmj14 ldl1-2 ldl2* DMRs. For both H3K4m2 and H3K4m3, the gain in histone methylation is greater in the triple demethylase mutant than *nrpe1-11* (P<4.4e-14, Mann-Whitney U test) despite *nrpe1-11* showing a greater reduction in CHH methylation (P<2.2e-16, Mann-Whitney U test).
(TIF)

Figure S6 Global H3K4m2/m3 ChIP analysis. Metaplots (A) and boxplots (B) of H3Km2 ChIP-seq read density (RPKM) over DMR groups in various RdDM mutant genotypes. For boxplots, DMRs were considered as the 1000 bp region extending +/−500 bp from the DMR midpoint. * indicates a significant gain in read density for a given library relative to wild type (P<1e-15, Mann-Whitney U Test) and ** indicates a gain in read density relative to all other libraries including wild type (P<1e-15, Mann-Whitney U Test). (C) and (D) present similar analyses for H3K4m3 ChIP-seq libraries with * representing a gain relative to wild type (P<1e-15, Mann-Whitney U Test) and ** representing a gain relative to wild type and all other libraries (P<4.4e-15, Mann-Whitney U Test).
(TIF)

Table S1 Genomic locations (TAIR10) of reduced CHH methylation DMRs.
(XLS)

Table S2 Primers and probes used in this study.
(XLS)
