# Supplementary figures and images for: Interplay between Active Chromatin Marks and RNA-Directed DNA Methylation in Arabidopsis thaliana
Source: PLoS Genet. 2013 Nov 7;9(11):e1003946. doi: 10.1371/journal.pgen.1003946 (PMC3820799; doi:10.1371/journal.pgen.1003946)

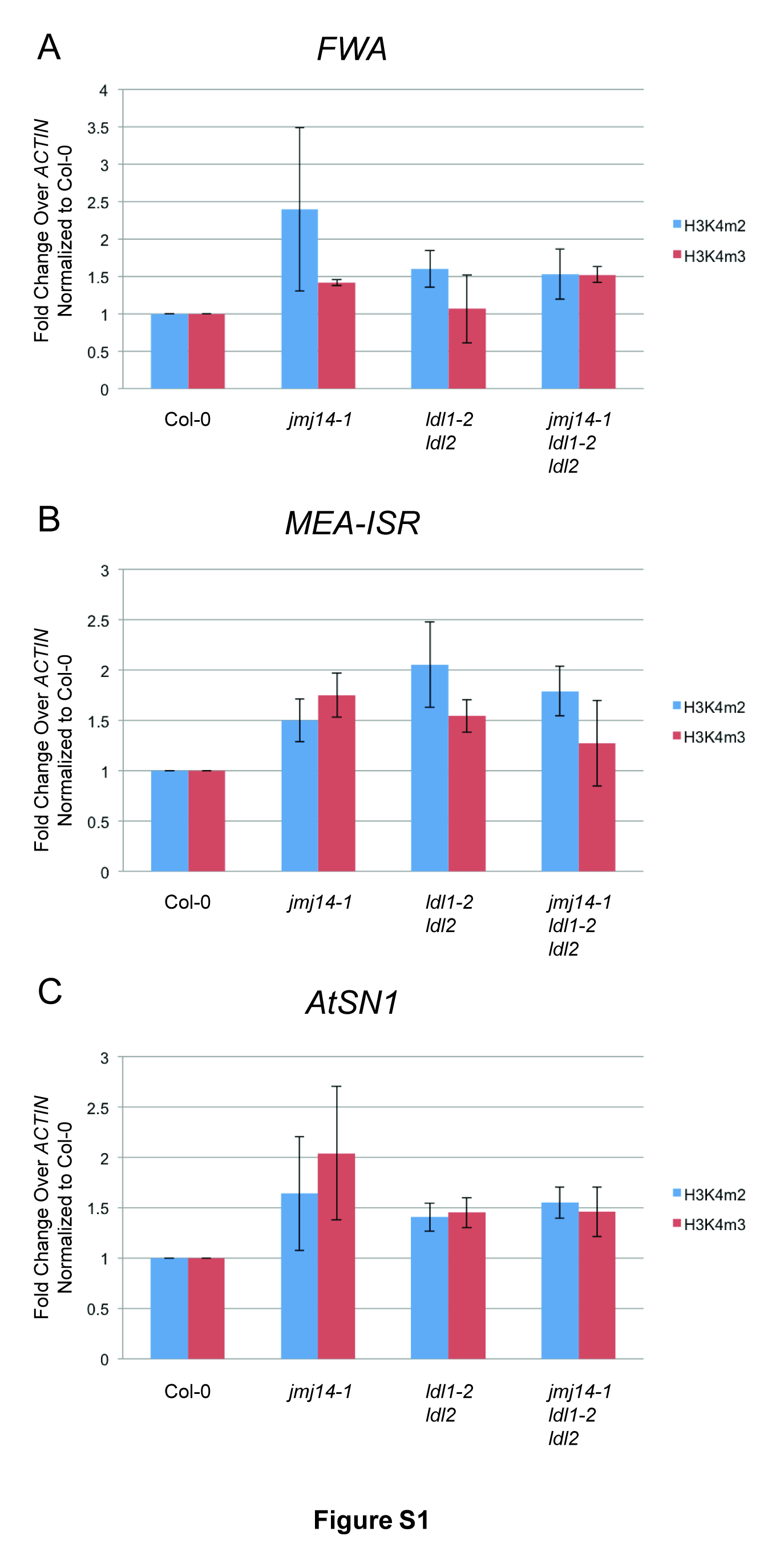

Supplement: Figure S1 — H3K4m2 and H3K4m3 ChIP-qPCR analysis of RdDM targets in histone demethylase mutants. The analysis was performed at FWA (A), MEA-ISR (B), and AtSN1 (C). Data were normalized to input DNA and to an internal control (ACTIN). The average of three independent ChIP-experiments is shown (for each experiment, qPCRs were performed in duplicate). (TIF) [file pgen.1003946.s001.tif]

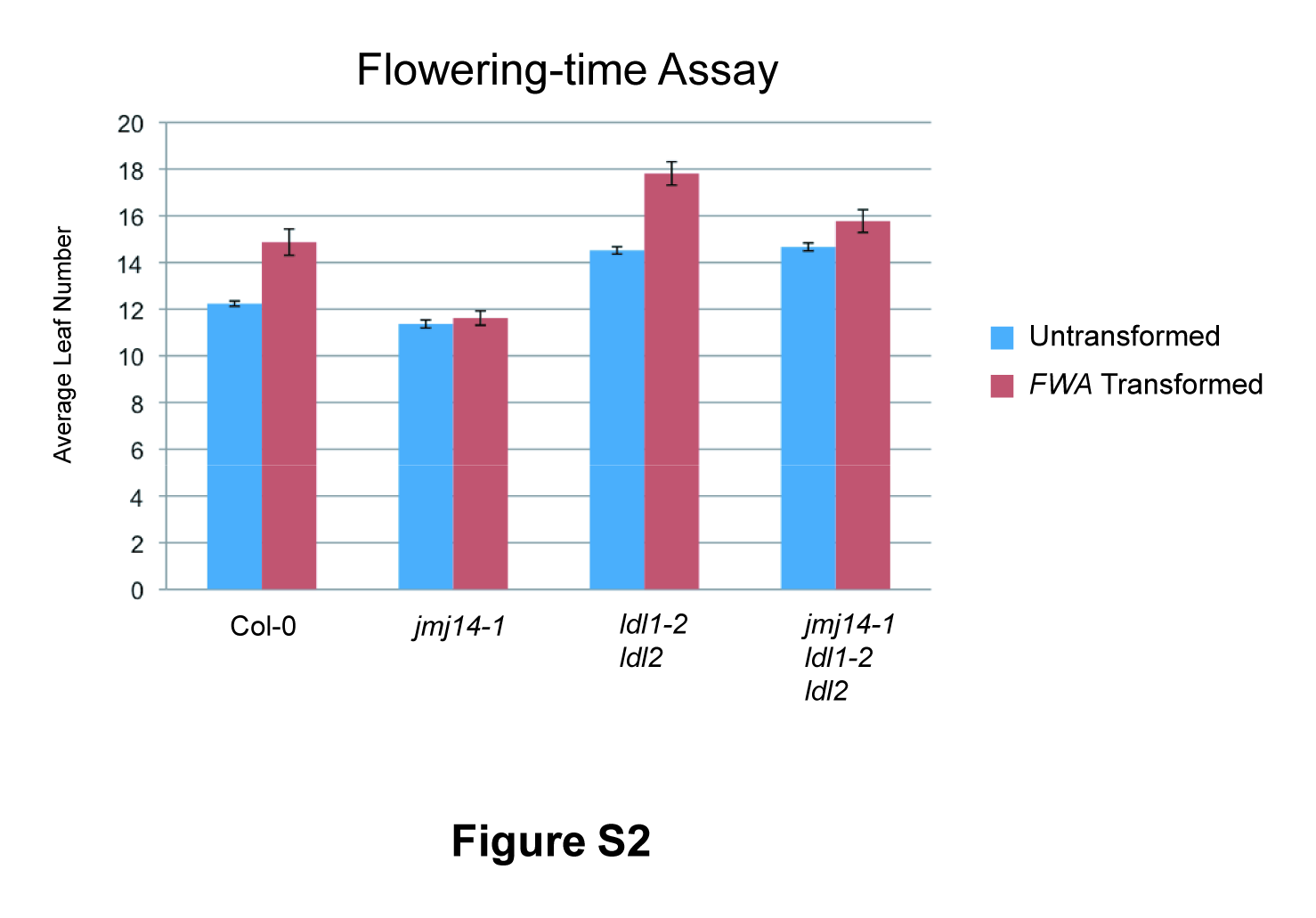

Supplement: Figure S2 — FWA methylation establishment assay and flowering-time analysis in chromatin effector mutants. Flowering-time is determined by the total number of rosette and cauline leaves when the first inflorescence appears. FWA transformed lines are compared to untransformed lines of the same genotype. The graph depicts averages from populations of >20 individual plants. (TIF) [file pgen.1003946.s002.tif]

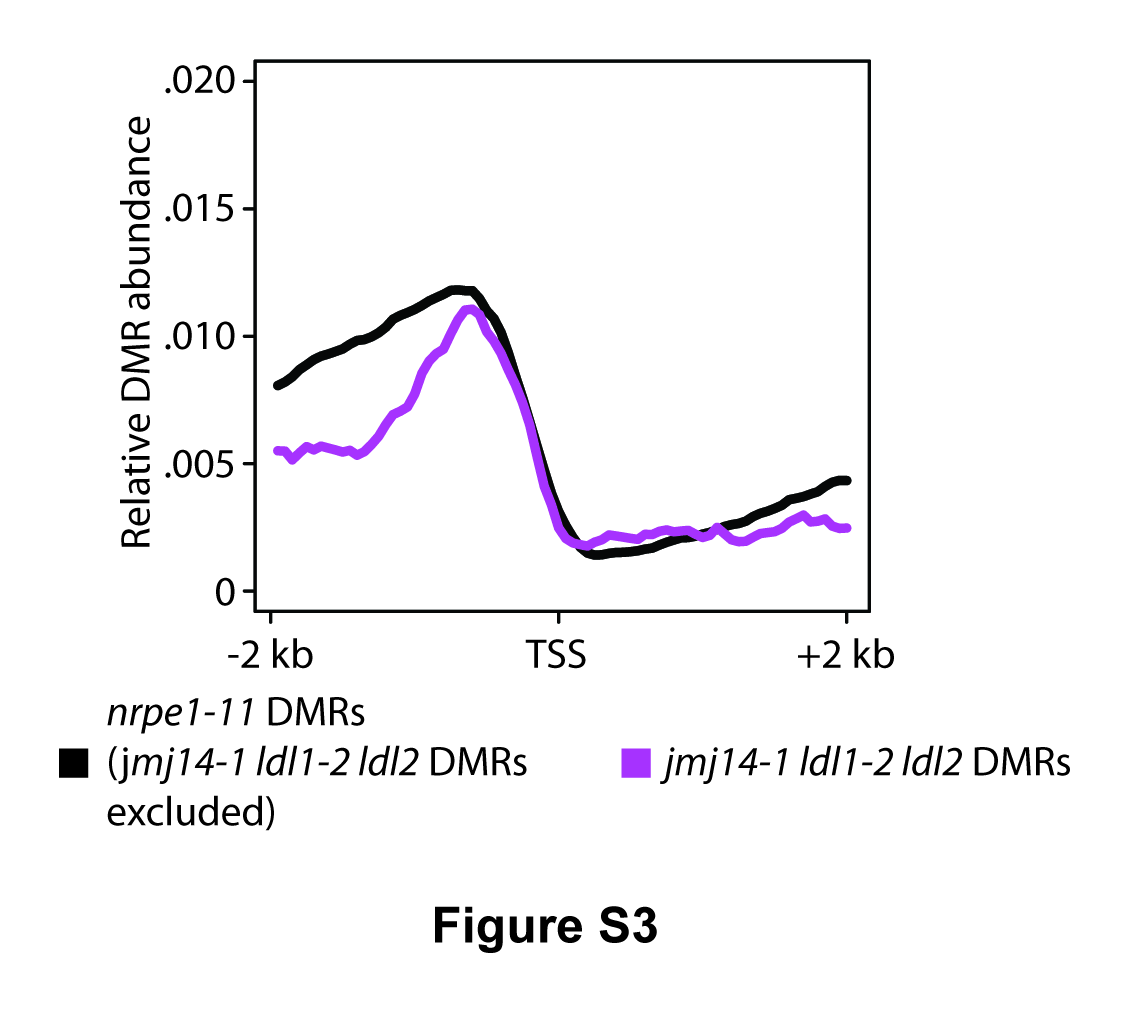

Supplement: Figure S3 — Relative abundance of mutant CHH DMRs at and around the transcription start site (TSS) of protein-coding genes. Relative abundance is calculated as ((average coverage of DMRs over gene region)/total number of thousands of DMRs). (TIF) [file pgen.1003946.s003.tif]

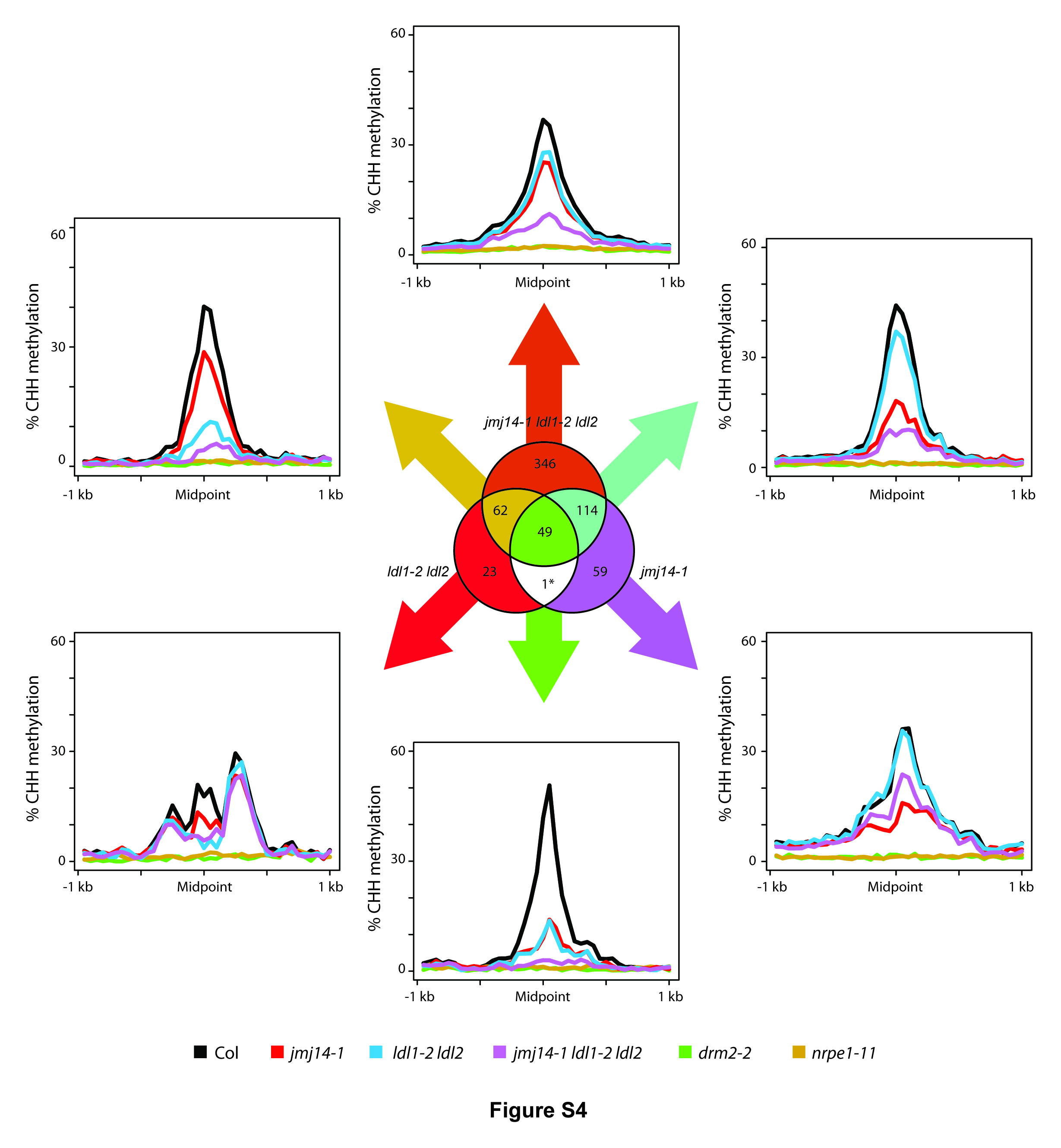

Supplement: Figure S4 — Analysis of subsets of DMRs defined in the demethylase mutants. Subsetting the demethylase DMRs identified in Figure 2A reveals some sites of preferential activity by either class of demethylase in regulating CHH methylation levels with a general trend of an enhanced CHH defect in the jmj14-1 ldl1-2 ldl2 triple mutant. * The single DMR represented as the union of jmj14-1 and ldl1-2 ldl2 DMRs to the exclusion of jmj14-1 ldl1-2 ldl2 DMRs was not plotted given the limited data in a single data point. (TIF) [file pgen.1003946.s004.tif]

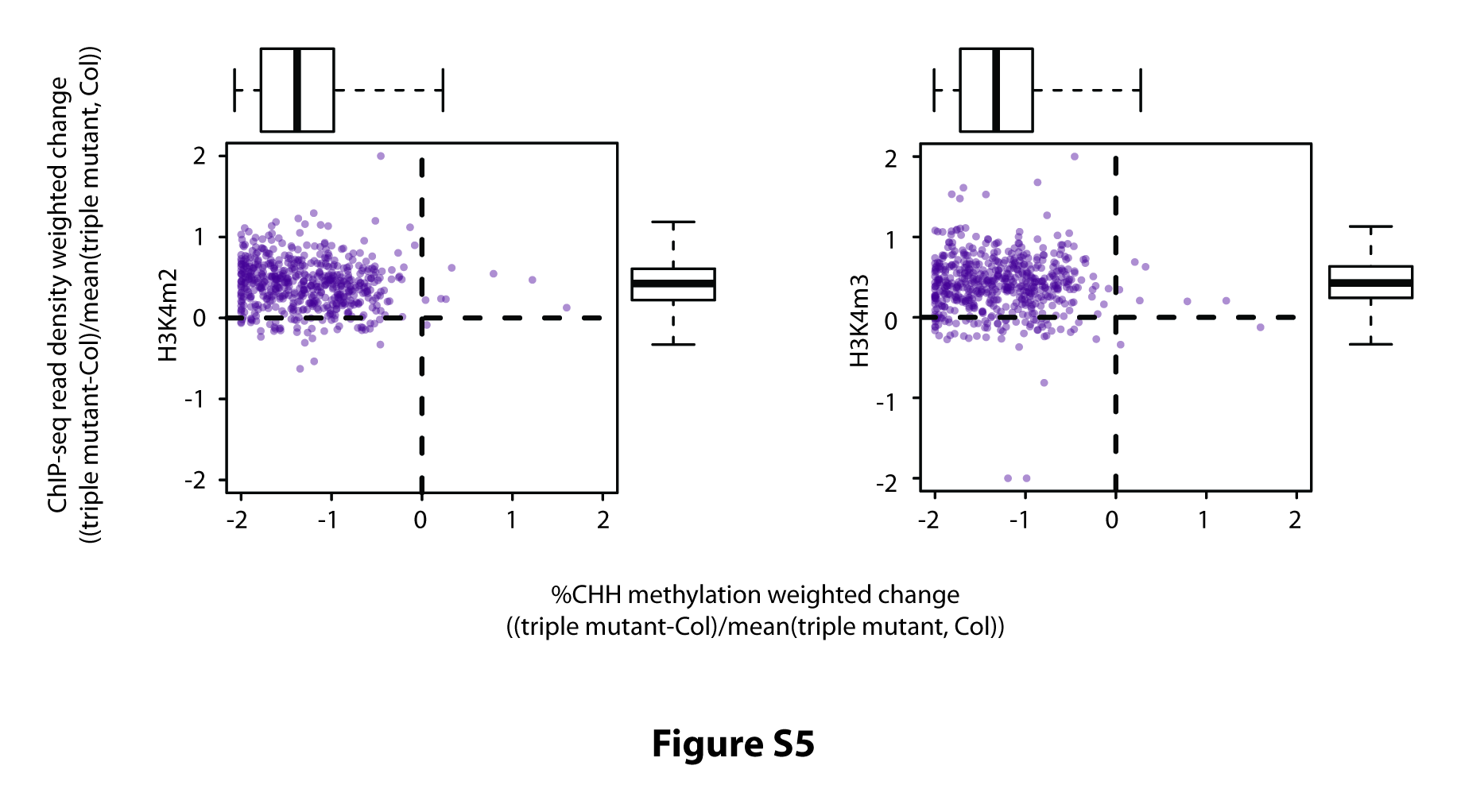

Supplement: Figure S5 — Correlation of H3K4 methylation changes and CHH context DNA methylation at jmj14-1 ldl1-2 ldl2 triple mutant CHH DMRs. Correlation of weighted change in H3K4m2/H3K4m3 and CHH methylation in jmj14-1 ldl1-2 ldl2 at jmj14 ldl1-2 ldl2 DMRs. For both H3K4m2 and H3K4m3, the gain in histone methylation is greater in the triple demethylase mutant than nrpe1-11 (P<4.4e-14, Mann-Whitney U test) despite nrpe1-11 showing a greater reduction in CHH methylation (P<2.2e-16, Mann-Whitney U test). (TIF) [file pgen.1003946.s005.tif]

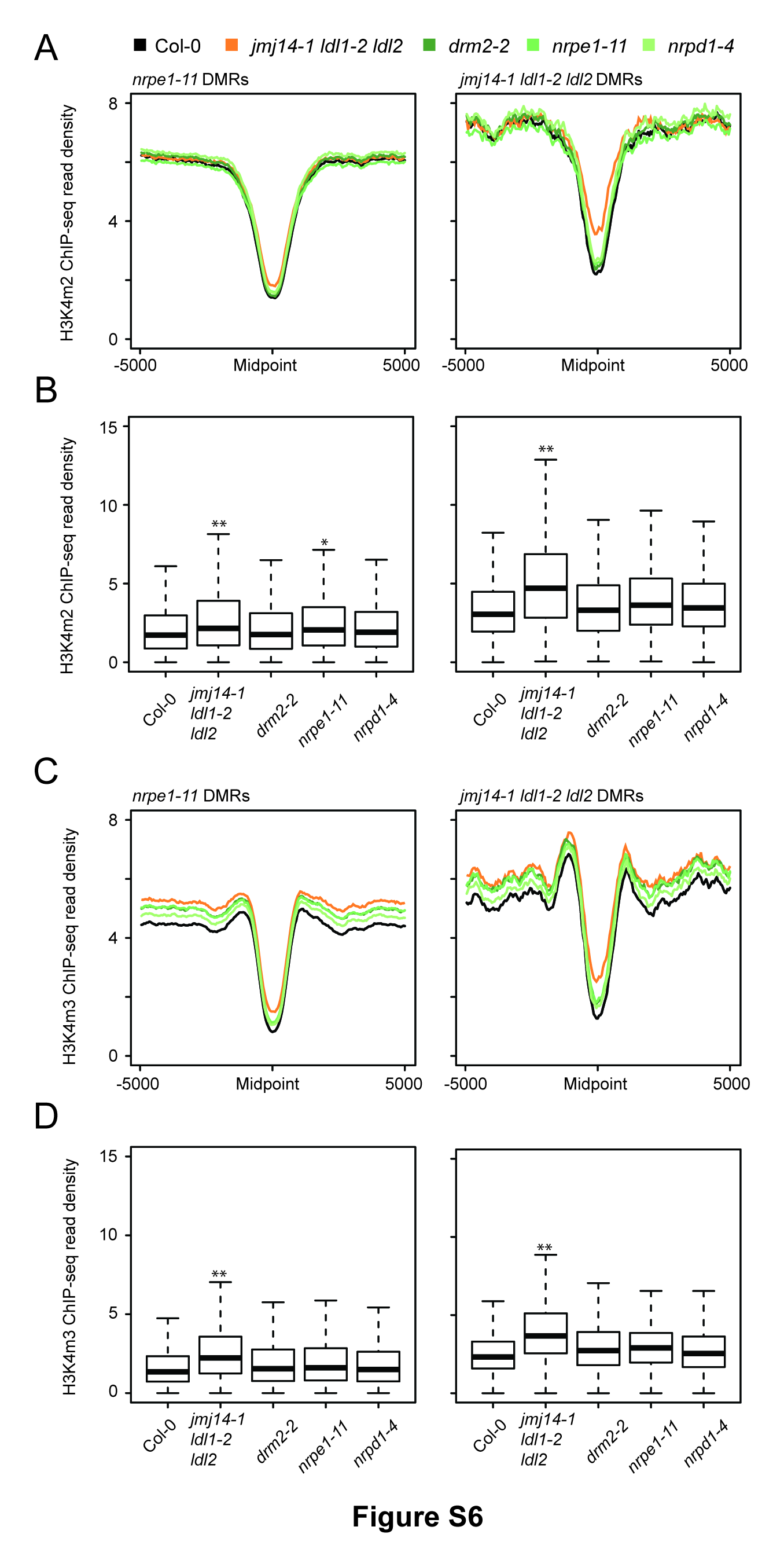

Supplement: Figure S6 — Global H3K4m2/m3 ChIP analysis. Metaplots (A) and boxplots (B) of H3Km2 ChIP-seq read density (RPKM) over DMR groups in various RdDM mutant genotypes. For boxplots, DMRs were considered as the 1000 bp region extending +/−500 bp from the DMR midpoint. * indicates a significant gain in read density for a given library relative to wild type (P<1e-15, Mann-Whitney U Test) and ** indicates a gain in read density relative to all other libraries including wild type (P<1e-15, Mann-Whitney U Test). (C) and (D) present similar analyses for H3K4m3 ChIP-seq libraries with * representing a gain relative to wild type (P<1e-15, Mann-Whitney U Test) and ** representing a gain relative to wild type and all other libraries (P<4.4e-15, Mann-Whitney U Test). (TIF) [file pgen.1003946.s006.tif]
